# The Black Unicorn Effect: Micro-daily Events and Satisfaction Decrease the COVID-19 Xenophobia, but Only for Those With Low Levels of Neuroticism

**DOI:** 10.1177/00332941231161278

**Published:** 2023-02-28

**Authors:** Ana Junça-Silva, Cristiana Vilela

**Affiliations:** Instituto Universitário de Lisboa (ISCTE-IUL), Business Research Unit (BRU-IUL), Lisboa, Portugal; Instituto Politécnico de Tomar (IPT), Tomar, Portugal; Instituto Politécnico de Tomar (IPT), Tomar, Portugal

**Keywords:** Neuroticism, daily micro-events, xenophobia, satisfaction, moderated mediation, black unicorn effect

## Abstract

Drawing on the behavioral concordance model and the trait activation theory, this study examined how and when daily micro-events influence COVID-19 xenophobic attitudes. First, we examined the mediating role of satisfaction, and then, tested the moderating role of neuroticism in the mediated relationship. Overall, 340 working adults volunteered to participate in this study. The findings revealed that (1) satisfaction mediated the negative relationship between daily micro-events and xenophobic attitudes and (2) neuroticism moderated this relationship such that xenophobic attitudes increased for neurotic individuals, even when their satisfaction increased. Our findings contribute to understanding the relationship between daily micro-events and COVID-19 xenophobia and provide empirical evidence for the combined effects of personality factors and affective factors on xenophobic attitudes. Furthermore, we evidence the existence of the black unicorn effect, that is, neurotic individuals tend to transpose their neurotic cognitions and emotions to xenophobic attitudes despite the uplifting and satisfying nature of positive events.

## Introduction

The COVID-19 pandemic crisis led to diverse social consequences, among them xenophobic attitudes and behaviors against foreigners, in particular those from China and East Asian ethnic origins (Cheng, 2020). Xenophobia is considered a hatred or fear of foreigners and involves essentially negative attitudes towards foreigners, being these characterized by disliking, fearing, or hating them (Harris, 2002). Indeed, there is evidence of an increase in xenophobia in the past two years (He, et al., 2021), specifically the xenophobic fears related to the idea that foreigners are spreading the COVID-19 virus – termed COVID-19 xenophobia (Taylor et al., 2020).

The affective events theory (AET; Weiss & Cropanzano, 1996) states that daily micro-events are affective events because are responsible for making individuals experience certain emotional reactions, such as satisfaction (e.g., Junça-Silva & Silva, 2022a, 2022). By making individuals emotionally react to them, daily micro-events also influence diverse attitudes and behaviors, such as xenophobic ones. Daily micro-events are the tiny little things that somehow irritate and annoy (daily hassles, e.g., having to deal with someone in a rotten mood) or please and gratify people (daily uplifts, e.g., being praised by someone) (Junça-Silva et al., 2021, 2022). Accordingly, we argue that daily micro-events will trigger affective feelings of satisfaction that may minimize COVID-19 xenophobia.

However, neuroticism – the tendency to be emotionally unstable by the recurrent experience of negative thoughts and emotions together with a low self-efficacy (Claridge & Davis, 2001; Myers & McGhee, 2010) – might moderate this path. The behavioral concordance model (Côté & Moskowitz, 1998) proposes that individuals with high scores on a given trait (e.g., neuroticism) tend to engage in behaviors concordant with that trait (e.g., fear of foreign and xenophobic attitudes). Moreover, the trait activation theory (Tett et al., 2021; Tett & Burnett, 2003) posits that personality traits are expressed as valued behaviors in response to trait-relevant situational cues; in other words, situational factors, such as daily micro-events (e.g., daily hassles) are triggers for the expression of congruent traits (e.g., neuroticism) which in turn may amplify or attenuate certain behaviors (e.g., xenophobic ones). Therefore, drawing upon both theories, we propose that neurotic individuals will present higher levels of xenophobic attitudes related to COVID-19, even when feeling satisfied with their day.

Although there are some studies demonstrating the relevance of situational factors accounting for xenophobic attitudes and behaviors (e.g., Esses & Hamilton, 2021), few researchers explored neuroticism as a boundary condition (moderating effect) under which individuals experiencing certain kinds of daily micro-events will engage in COVID-19 xenophobic attitudes. Therefore, this study aimed to (1) analyze the mediating role of satisfaction on the relationship between daily micro-events and COVID-19 xenophobia and; (2) test the moderating role of neuroticism in this mediating path.

## Theoretical framework and hypotheses development

### The Relationship Between Daily Micro-Events and COVID-19 Xenophobia

The COVID-19 pandemic crisis has led, not only to health-damaging consequences but also to social ones (Taylor et al., 2020). Xenophobia has increased significantly since early 2020, the date on which the coronavirus began to spread all over the world (He et al., 2021). From this date on, xenophobic attitudes toward foreigners increased all over the world (Newbold, 2020).

Xenophobia is defined as a hatred or fear of foreigners and involves essentially negative attitudes towards foreigners, being these characterized by disliking, fearing, or hating them (Harris, 2002). With the rapid spread of the COVID-19 virus, its related xenophobia emerged as a fear that foreigners are sources of the virus (Taylor et al., 2020). Indeed, the threat of a public health emergency, along with other social-economical and organizational changes, such as job loss, or the workers’ lay-off, enhanced the fear of individuals which, therefore, catalyzed underlying xenophobic feelings and attitudes towards foreigners. As Murray and Marx (2013) noted, feeling threat predicts xenophobia and prejudice toward foreigners. Furthermore, when something like a public health emergency happens, individuals are more likely to feel a generalized lack of control of situations, which is positively related to xenophobic attitudes and intensifies the xenophobic reactions to perceived threats or uncertain situations (Greenaway et al., 2013; Harrell et al., 2017).

Likewise, daily micro-events, the tiny things that happen in individuals’ life at work (Junça-Silva et al., 2021) might help to explain how xenophobic attitudes arise. The affective events theory (Wang, Zhu, Dormann, Song, & Bakker, 2020) has explored these events and proposes that the work environment, in which the individual spends most of the day, promotes the occurrence of these events. Then, these events trigger affective reactions (e.g., satisfaction) that, in turn, influence attitudes and behaviors (such as xenophobia, and bullying, among others; Glasø et al., 2011). Furthermore, the Aet also states that individual dispositions (mood and personality) may buffer or intensify the affective and attitudinal reactions to daily micro-events.

Daily micro-events are divided into positive (daily uplifts) and negative (daily hassles). Daily uplifts are the daily micro-experiences that are appraised as positive and are seen as uplifts of individuals’ well-being (Ohly & Schmitt, 2015). Examples of daily uplifts may include when someone is praised for something s/he has done, or when s/he makes meaningful breaks from work during the day. On the other hand, daily hassles have the opposite effect, that is, not only are threatening to the individuals’ goals and well-being but are also a catalyst for negative daily behaviors (Junça-Silva et al., 2021). Examples of daily hassles are having to deal with someone in a rotten mood or being interrupted while performing a task.

### The Mediating Role of Satisfaction

As proposed by the AET, daily micro-events may trigger affective reactions by making individuals feel a certain way after the micro-episode. For instance, an individual may feel satisfied after being praised for the work done or may feel dissatisfied by having failed some deadline. Moreover, some studies have demonstrated that the ratio of daily micro-events (that is, the proportion of daily uplifts compared to daily hassles) is a significant predictor of individuals' level of satisfaction at the end of the day (e.g., Junça-Silva et al., 2022), well-being (e.g., Landolfi et al., 2022) and affect ratio (e.g., Christensen et al., 2022; Junça-Silva & Silva, 2022a, 2022).

Empirically, the relationship between daily micro-events, affect and behaviors are well-documented in the literature (e.g., Glasø et al., 2011; Ohly & Schmitt, 2015). For instance, a daily diary study demonstrated that two kinds of daily events (recognition and achievement) predicted work engagement via daily satisfaction (Wang, Zhu, Dormann, Song, & Bakker, 2020). Similarly, Reis et al. (2018), in a study conducted over two weeks, evidenced that daily social interactions positively influenced individuals’ well-being via their daily satisfaction. Plus, Gable et al. (2018) showed that positive events were linked to higher satisfaction and, as a result, overall well-being. More recently, Ågotnes, et al. (2021), in a diary study with naval cadets, evidenced that daily hassles predicted bullying-related negative attitudes. In a similar vein, Junça-Silva, et al. (2022), demonstrated that both daily hassles and uplifts were antecedents of daily satisfaction and contextual work behaviors. Landolfi and colleagues (2022) also evidenced that daily events related to work-family conflict predicted affect and attitudes toward work and family.

To date, no studies have explored the role of daily micro-events for xenophobic attitudes, in particular, in these emergency times, such as the COVID-19 pandemic crisis. Despite that and based on the AET and the empirical findings summarized above; we defined the following hypothesis.

**H1.** Satisfaction mediates the negative relationship between the ratio of daily micro-events and COVID-19 xenophobia.

### The Moderating Role of Neuroticism

As mentioned earlier, the AET states that certain personal dispositions, such as personality traits, attenuate or intensify the reactions to micro-daily events. Neuroticism is a trait that is linked to the experience of frequent negative affect, anxiety, fear, emotional instability (Jeronimus et al., 2016; McCrae & Costa, 1999), and decreased mental health (Junça-Silva & Silva, 2022a, 2022), is one of the more investigated 5 traits (extraversion, openness, agreeableness, and consciousness) described by the Big-5 personality theory (Costa & McCrae, 1992).

We opted for neuroticism because research has evidenced that neurotic individuals tend to react more intensely to pandemic crises and with dysfunctional affective reactions (e.g., fear) and attitudes (e.g., xenophobia) (e.g., Taylor et al., 2020). This may be explained by the behavioral congruence model (Côté & Moskowitz, 1998), individuals should experience greater positive affect and less negative affect in situations (pandemic crises) that are congruent with their personality characteristics (emotional instability).

Neuroticism is defined by the tendency to (1) view and appraise the world in a black manner, together with (2) a low self-efficacy regarding the ability to deal with unexpected, negative, or stressful events, leading to (3) intense affective and attitudinal responses (Barlow, et al., 2014).

Diverse studies have consistently demonstrated that neuroticism is associated with depression (Jeronimus, et al., 2016). Furthermore, individuals who score high on neuroticism tend to be worried about everything, anxious, moody, tense, and easily distressed (Watts, et al., 2019). A recent study conducted by Junça-Silva & Silva, 2022a, 2022) emphasized that neurotic individuals – “individuals who live the life without unicorns” (pp. 1) tend to see the world through a black veil. Moreover, neuroticism has been associated with higher perceived uncertainty, frequent feelings of guilt and anger, and aggressive behaviors (Sun et al., 2016). Likewise, neurotic individuals tend to act more impulsively, when compared to those who score lower (Mitchell et al., 2021). Thus, we may conclude that neuroticism has a volatile and negative nature, as those who score high in this trait, more frequently and intensely experience, negative emotions, which are associated with the lack of control in response to stressful situations or environments, such as this pandemic crisis of the COVID-19.

The trait-activation theory (Tett & Burnett, 2003) supports these findings as it stated that personality traits can influence behavior by providing trait-relevant situational cues. Inversely, a constraint or stressful situation can inhibit trait-relevant behavioral expression by limiting situational cues. That is, specific behaviors related to some traits (in this case, neuroticism) are activated based on the appraisal of the events and situations in which individuals are involved, and on the inherent gains of it. In this case, neurotic individuals may activate the COVID-19 xenophobic attitudes to reduce their anxiety and perceive a lack of control triggered by the perceived uncertain context of the COVID-19 pandemic crisis. In other words, xenophobic attitudes might be activated as a strategy to recover from the negative situations perpetuated by the coronavirus.

**Figure 1.**
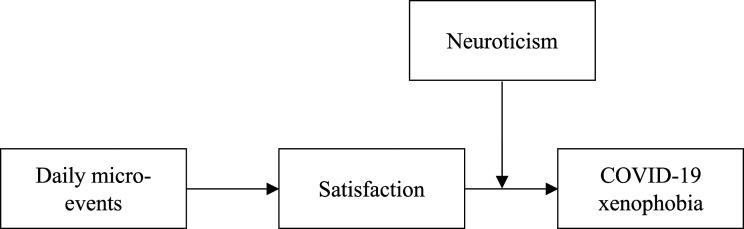
The hypothesized model.

In addition, based on the behavioral congruence model, it is likely that people high in neuroticism, by feeling vulnerable and perceiving uncertainty in the context in which they are living, will tend to have more COVID-19 xenophobic attitudes; this will lead to the feeling of congruency between attitudes, behavior and their personality (Côté & Moskowitz, 1998). Thus, using the trait activation theory, and the behavioral congruence model, we investigated the role of neuroticism as an important boundary condition affecting the relationship between micro-daily events, satisfaction, and COVID-19 xenophobic attitudes. Therefore, we defined the following:

**H2.** Neuroticism moderates the indirect relationship between the ratio of daily micro-events and COVID-19 xenophobia via satisfaction, such that the relationship will be weaker for higher levels of neuroticism (vs. lower) (Figure 1).

## Method

### Participants and Procedure

Overall, we recruited a total of 340 adult participants, from which 73.3% were female. The mean age was 38.24 years old (*SD* = 13.5), and the mean tenure was 10.37 years (*SD* = 10) On average, individuals worked 39.4 hours per week (*SD* = 14.58). Most participants had their high school complete (44%) followed by those who held graduation (40%). The majority reported living in a low socioeconomic status (58.4%), followed by those who reported living in a high socioeconomic status (33.5%). Only 55% of the sample had children (see Table 1 for a synthesis).

**Table 1.** Sample Characteristics.

Variable	*%*	*M (SD)*
Female	73.3%	—
Male	26.7%	—
Higher education degree	40%	—
High school complete	44%	—
Telework	21%	—
Face-to-face work	79%	—
With a supervisor role	29%	—
Low socioeconomic status	58.4%	—
High socioeconomic status	33.5%	—
Having children	55%	—
Age	—	38.24 (13.5)
Tenure	—	10.37 (10)
Weekly working hours	—	39.4 (14.58)

*Note. N* = 340.

We contacted participants, via email, from our professional networks and asked them to participate in a study about attitudes and stress. Those who agreed to participate received a second email asking them to sign an informed consent and explaining that the study was anonymous and confidential. Moreover, we also explained that they could quit the study at any time if they intended to. In that email, was also sent the hyperlink to the survey. We collected data between January and March 2021, in the second mandatory confinement due to the COVID-19 virus, in Portugal. From the 350 contacts we have made, we obtained 340 valid responses (response rate = 97%).

### Measures

#### Daily Micro-Events

We used the 18-item scale for daily hassles and uplifts at work (SDHUW; Junça Silva et al., 2020). It measured daily hassles (10 items, e.g., “Today, I had to deal with someone in a rotten mood”) and uplifts (eight items, e.g., “Today, I helped someone”). Responses were made on a 5-point Likert scale (1 – *never*; 5 – *4 times or more*). For the daily hassles’ dimension, the reliability was α = .83 and Ω = .83, and for the daily uplifts’ dimension was α = .89 and Ω = .88.

#### Satisfaction

We assessed satisfaction with three items from Junça-Silva and colleagues (2021) (e.g., “Today, my day was very good”) that evaluated the participants’ perception of that day on a 5-point Likert Scale (1 = *totally disagree*; 5 = *totally agree*). Cronbach’s alpha was .84 and McDonald’s Omega Coefficient was .85.

#### COVID-19 Xenophobic Attitudes

We used 6 items from the COVID-19 Stress Scales (Taylor et al., 2020) to measure COVID-19 xenophobia (e.g., “I am worried about coming into contact with foreigners because they might have the virus”). Responses were given on a 5-point Likert scale (1 – *not at all*; 5 – *extremely*) (α = .93 and Ω = .93).

#### Neuroticism

To measure neuroticism, we used four items from the Mini-IPIP scales (Donnellan et al., 2006). Responses were given on a 5-point Likert scale (1 - *very inaccurate*; 5 - *very accurate*) (e.g., “I have frequent mood swings”; α = .50 and Ω = .77).

#### Control Variables

We used sex and age as control variables, as these variables may account for differences in daily experienced satisfaction (Dello Russo et al., 2021). Sex may influence satisfaction because men and women have different affective patterns; for instance, women tend to react more intensely to daily micro-events, whereas men tend to be less vulnerable to these situational influences (Junça-Silva et al., 2022). In addition, age may influence satisfaction because as people get old, they tend to adapt their reactions more effectively, so they also easily achieve satisfaction, whereas youngers tend to be more impulsive, and as such their affective reactions tend to be more intense, less adaptive and more volatile to situational influences (Dello Russo et al., 2021; Junça-Silva et al., 2022).

### Data Analyses

To test our hypotheses, we created a ratio between daily uplifts and daily hassles. This ratio allowed us to identify the proportionality of daily uplifts regarding daily hassles. When the ratio is higher than one, it means that daily uplifts occurred more frequently than daily hassles did (Junça-Silva et al., 2022).

Then we calculated the descriptive statistics, correlations, and reliabilities with IBM SPSS Statistics, version 27. To test our hypotheses, we used the PROCESS macro (Hayes, 2018), specifically, we used model four to test the first hypothesis (mediation hypothesis) and model 14 to test the second one (moderated mediation hypothesis). We used bootstrapping method (5000 bootstrap samples) with 95% confidence intervals (CIs) to test the model's significance. The 95% of CIs that did not include zero indicated a significant effect.

Because both the predictor (daily micro-events) and the criterion variable (COVID-19 xenophobia) were measured at the same time, we followed some strategies to prevent the issue of common method variance (Junça-Silva et al., 2022; Podsakoff et al., 2012). First, we shuffled the questions of various measures and then used some dummy questions non-related to the main research goal (e.g., I do not like sunny days). Second, we used Harman’s single-factor test to assess the common method variance. The results from it showed that the single factor accounted for only 19.30% of the variance, which was clearly below the threshold value of 50% proposed by Podsakoff et al. (2012); hence, the common method variance issue was not severe for this study.

## Results

### Confirmatory Factor Analyses

Before testing the research hypotheses, we performed four confirmatory factor analyses (CFA) to confirm the independence of the main variables of the study by using the software JASP version .14.1. To do so, we used a combination of fit indices – comparative fit index (CFI), Tucker–Lewis index (TLI), standardized root mean square residual (SRMR) and root mean square error of approximation (RMSEA) – to assess the adequacy of the model and compared the hypothesized model with several reasonable alternative measurement models (Bentler & Bonett, 1980). When the CFI and TLI scores are above .88 and the SRMR and RMSEA are below .07 it is assumed to be a model with a good fit to the data (Hair et al., 2010).

As such, we estimated four alternative models. Model 1 was the hypothesized four-factor model comprising separate scales for daily micro-events, satisfaction, COVID-19 xenophobia, and neuroticism. Model 2 was a three-factor model where daily micro-events and satisfaction were combined into a unique factor. Moderjran alternative three-factor model where neuroticism and COVID-19 xenophobia were combined into a single factor. Model 4 was a one-factor solution in which all items were loaded onto a single factor. Table 2 shows that our hypothesized model (Model 1) provided a good fit for the data (CFI = .89, TLI = .88, SRMR = .09, and RMSEA = .06). Moreover, all other alternative models showed a poorer fit compared to the fit of Model 1. These results together with the Cronbach alpha reliability, MacDonald’s Omega coefficient, Harman’s single factor test, and the scores across all the measurement scales evidenced the discriminant and convergent validity of the study; hence, we proceeded with the test of hypotheses.

**Table 2.** Confirmatory Factor Analyses: Model Fit Indices.

Measurement Model Comparison	SRMR	CFI	TLI	RMSEA
Model 1 (4-factor model: DME, satisfaction, COVID-19 xenophobia, and neuroticism)	.09	.89	.88	.06
Model 2 (3-factor model: COVID-19 xenophobia, neuroticism and DME and satisfaction merged)	.14	.63	.61	.11
Model 3 (3-factor model: COVID-19 xenophobia and neuroticism merged, satisfaction and DME)	.13	.69	.66	.10
Model 4 (1-factor model: All measures loaded on a single latent factor)	.19	.33	.28	.15

*Note. N* = 340; SRMR = standardized root mean square residual; CFI = comparative fit index; TLI = Tucker–Lewis index; RMSEA = root mean square error of approximation. DME = daily micro-events.

### Descriptive Statistics

Table 3 presents the descriptive statistics and the correlations among the variables. As we can see, daily micro-events showed a positive and significant relation with satisfaction but not with COVID-19 xenophobia or neuroticism. In addition, satisfaction was positively related to xenophobia but not to neuroticism, and this one did not present any significant association with none of the variables.

**Table 3.** Descriptive Statistics, Correlations and Cronbach’s Alphas.

Variable	*molar*	*SD*	1	2	3	4
1. Daily-micro events	2.75	3.94	(.86)			
2.COVID-19 xenophobia^*1*^	2.06	1.07	.04	(.93)		
3. Satisfaction^1^	3.07	.88	.33**	.06*	(.84)	
4. Neuroticism^*1*^	2.99	.54	.02	.01	−.01	(.50)

*Note. N* = 340; ^*^p < .05 ^**^p < .001. Cronbach’ alfas are in brackets.

### Hypotheses Testing

#### Mediation Hypothesis

The first hypothesis stated that satisfaction would mediate the negative relationship between daily micro-events and COVID-19 xenophobia. The findings demonstrated that a higher ratio of daily micro-events significantly influenced higher satisfaction (*B* = .09, *p* < .001, CI 95% [.04, .10]) (see model 1 of Table 2). When satisfaction entered the model, daily micro-events were no longer significantly influencing COVID-19 xenophobia (*B* = .01, *p* > .05 CI 95% [-.03, .03]) (see model 2 of Table 4), moreover the bootstrapping indicated that the mediation effect of satisfaction was significant (*B* = .03, *SE* = .01, CI95% [.00, .03]), evidencing a full mediating effect of satisfaction. The Sobel test-associated statistic was 1.61 (*p* < .05), thus, lending support to the first hypothesis.

**Table 4.** The Mediation Effect of Satisfaction on the Relationship Between Daily Micro-Events and COVID-19 Xenophobia.

	Model 1 (Satisfaction)		Model 2 (COVID-19 Xenophobia)	
Predictor	*B*	*t*	*B*	*T*
Daily micro-events	.09**	5.34	.01	.16
Satisfaction	—		.12	1.28
Age	−.00	−.94	−.00	−.22
Sex	−.02	−.15	−.18	−1.15
*R* ^2^	11.48		.02	
F	10.06**		.86*	

*Note. N* = 340; ^*^p < .05 ^**^p < .001.

#### Moderated Mediation Hypothesis

The second hypothesis stated that neuroticism would moderate the mediating path from daily micro-events to COVID-19 xenophobia via satisfaction. First, we tested the simple moderation (with model 1 of the macro-PROCESS) as recommended by Hayes (2018). The results showed that the interaction effect between satisfaction and neuroticism was significant (*B* = .32, *p* < .05, CI 95% [.04, .61]). Then, we proceeded with the analysis of the moderated mediation model (model 14).

**Figure 2.**
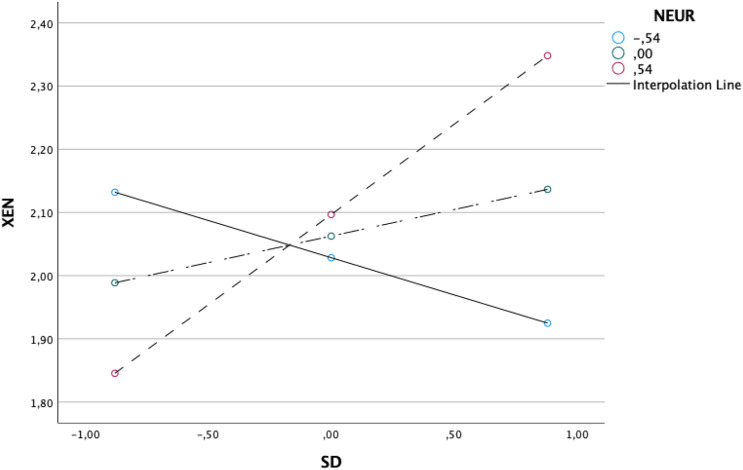
The moderation effect of neuroticism on the relationship between micro-daily events and COVID-19 xenophobia via satisfaction.

**Figure 3.**
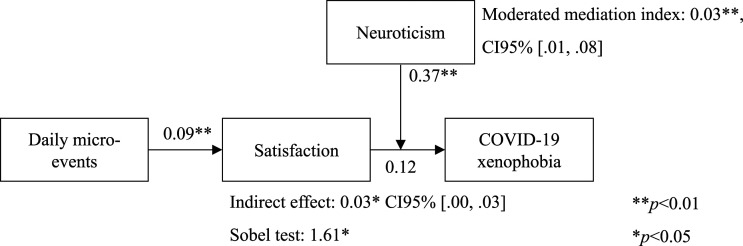
The overall model with the results.

The moderated mediation index was significant (*B* = .03, *SE* = .02, CI 95% [.01, .08]) (see Table 5). The simple slope analysis showed that the indirect effect was conditional upon the levels of the moderator (neuroticism), specifically when the moderator presented higher levels (+1SD: effect = .02, *SE* = .02, CI95% [.01, .07]). The indirect effect was no longer significant when the moderator presented lower (-1SD: effect = −.01, *SE* = .01, CI95% [-.03, .01]) and mean levels (M: effect = .01, *SE* = .01, CI95% [-.00, .03]) (see Figure 2). As we can see from Figure 2, the indirect effect of daily micro-events on COVID-19 xenophobia via satisfaction was stronger for those who scored higher on neuroticism. On the other hand, even though no longer significant, the indirect effect was buffered for those who scored lower on neuroticism. Thus, the second hypothesis received support. See, Figure 3 for a synthesis of the results (Table 6).

**Table 5.** The moderated mediation effect analysis.

	Model 1 (Satisfaction)		Model 2 (COVID-19 Xenophobia)	
Predictor	*B*	*t*	*B*	*T*
Daily micro-events	.09**	5.43	−.00	−.07
Satisfaction	—	—	.12	1.00
Neuroticism	—	—	.06	.49
Satisfaction*Neuroticism	—	—	.37**	2.47
Age	−.00	−.94	−.00	−.22
Sex	−.02	−.15	−.18	−1.15
*R* ^2^	11.03		.03	
F	29.50**		1.83*	

*Note. N* = 340; ^*^p < .05 ^**^p < .001.

**Table 6.** Bootstrap results for the moderated mediation effect.

	Neuroticism	Effect	Boot SE	LLCI	ULCI
Conditional indirect effects	-1SD	−.01	.01	−.03	.01
	M	.01	.01	−.00	.03
	+1SD	.02	.02**	.01	.07

*Note. N* = 340; ^*^p < .05 ^**^p < .001.

## Discussion

This study uses a sample of working adults to test whether (1) satisfaction mediates the negative link from daily micro-events to COVID-19 xenophobia, and; (2) neuroticism moderates this mediating path.

To the best of our knowledge, no study has explored the influence of such events on xenophobic attitudes. Thus, this study contributes to expanding the literature on such topics, by demonstrating that daily situational factors influence the individuals’ xenophobic fears triggered by the COVID-19 virus.

First, the findings are in line with the AET by demonstrating that daily micro-events positively influence satisfaction that, in turn, decreases COVID-19 xenophobia. However, this relationship is moderated by the levels of neuroticism, as this overshadows the beneficial effects of having a good and satisfactory day by leading to more COVID-19 xenophobic attitudes.

### Theoretical Implications

Overall, this research highlights the relevant role of daily micro-events experienced in the work context as a trigger or a limiter for xenophobic-related attitudes via experienced satisfaction. In other words, when individuals experience a positive ratio of daily micro-events (more daily uplifts than daily hassles), they tend to feel more satisfied with their day which appears to minimize their COVID-19 xenophobic attitudes and fears. Hence, a day full of daily uplifts (e.g., being praised for something done, or making meaningful breaks during the day to recover from work) may not only lead workers more satisfied but also limit their xenophobic attitudes, which is particularly important given that xenophobic behaviors and hate crimes against outgroups (e.g., Anti-Asian Racism) have risen as one critical global issue during the COVID-19 pandemic (Reny & Barreto, 2022). On the opposite, a day with frequently experienced daily hassles (e.g., having to deal with someone aggressive or in a rotten mood) may decrease the feelings of daily satisfaction and trigger xenophobic attitudes toward individuals from outgroups.

The AET has a well-documented history, as there is plenty of evidence of the paths from daily hassles and uplifts to several attitudes and behaviors via affective reactions (e.g., Junça-Silva et al., 2021; Ohly & Schmitt, 2015). For instance, Vie et al. (2010), demonstrated that micro-aggressions influenced affective reactions (anxiety and anger) that, in turn, decreased individuals’ well-being. Recently, Junça-Silva et al. (2022), demonstrated that both daily hassles and uplifts were antecedents of daily satisfaction and contextual work behaviors, and Ågotnes et al. (2021), in a diary study with naval cadets, showed that daily hassles predicted bullying-related negative attitudes.

The mediating relationship appears to be conditional upon the levels of neuroticism. Neuroticism is the tendency to see life with a black veil (Junça-Silva & Silva, 2022a, 2022) and to experience negative affective experiences (Barlow et al., 2014). This study reveals that the mediating path from daily micro-events to COVID-19 xenophobia via satisfaction occurs differently according to the individuals’ levels of neuroticism. That is, a positive ratio of daily micro-events makes individuals feel satisfied with their day (Junça Silva et al., 2020, 2017, 2021), however, if these individuals have neurotic tendencies, then they tend to have more xenophobic attitudes, despite the positivity of the day. This makes neuroticism an important boundary condition to understanding when and for whom COVID-19 xenophobic fears are likely to occur. Thus, even when neurotic individuals have a good day, they tend to transfer their black thoughts and concerns to individuals from the out-group, leading therefore to xenophobic fears and attitudes towards them.

The behavioral congruence model supports this finding. Accordingly, the relationship between daily micro-events, satisfaction, and COVID-19 xenophobic attitudes is intensified for individuals who score high in trait-neuroticism (congruence between personality traits and attitudes), because it will lead to the feeling of congruency between attitudes, behavior, and personality (Côté & Moskowitz, 1998).

Furthermore, the trait-activation theory (Tett & Burnett, 2003) is in line with these findings as it states that personality traits can influence attitudes by providing trait-relevant situational cues. Accordingly, neurotic individuals may engage in COVID-19 xenophobic attitudes as a strategy to reduce their anxiety and lack of control triggered by the perceived uncertain context of the COVID-19 pandemic crisis. In addition, a negative and thereby unsatisfactory day – full of daily hassles – may also give them the necessary cues to activate their neurotic trait, and then intensify their xenophobic attitudes toward the outgroup.

Empirically, there is also some support for the role of neuroticism as a boundary condition of diverse attitudes; for instance, Tong (2010) evidenced that neuroticism affects, not only how individuals appraise their contexts, but also the reactivity of their negative emotions to appraisals. Similarly, Oh and Tong (2020) demonstrated that neuroticism moderated the link between negative emotion differentiation and health, in such a way that for individuals low on neuroticism, negative emotion differentiation was a beneficial regulatory capacity with significant positive associations with health, but this did not occur for individuals higher on neuroticism. More recently, Junça-Silva & Silva, 2022a, 2022), in their diary study, demonstrated that the relationship between daily uncertainty and mental health via negative affect was intensified by neuroticism. Furthermore, the authors emphasized the relevance of neuroticism as a boundary condition for daily life routines and its related outcomes.

Thus, neuroticism might be understood as *the black unicorn effect*, once it enhances the blackness of the pandemic environment in which we are living. We may say that neuroticism threatens the uplifting nature of positive events, turning them into xenophobic attitudes.

### Limitations and Future Directions

This study has four limitations to consider. First, the self-reported nature of the data may lead to common method bias. Second, the cross-sectional design may bias our conclusions. In this sense, future studies would test the model with other designs, such as longitudinal or diaries. Diary studies help to capture the dynamics of daily life and is, therefore, suitable to explore variables such as daily micro-events and satisfaction (Ohly & Schmitt, 2015). Third, most of the sample was female which may have biased the results. For instance, some studies have shown that women tend to be more neurotic and impulsive (Knežević, 2018) than men. Thus, in future research, a balanced sample of men and women should be used to test this model and analyze whether these effects remain the same. Fourth, we must consider that data was gathered during the second mandatory confinement of this pandemic crisis which may have influenced the reports. At this stage, most individuals were in different conditions – social isolation - as the usual conditions of daily life. Therefore, a new test of the model, out of the pandemic mandatory confinement would be conducted to understand whether these effects are maintained. As some studies reported, mandatory confinements were an additional source of negative affective experiences and perceived uncertainty (e.g., Junça-Silva & Silva, 2022a, 2022; Taylor et al., 2020) which may have intensified not only for these results but also for the increased levels of COVID-19 xenophobia to the outgroups (Reny & Barreto, 2022).

Despite the limitations, this study opens avenues for further research. First, future studies would test the moderating role of other personality characteristics – for instance, the other Big-5 traits – as a way to understand other boundary conditions, that could buffer or intensify, the path from daily micro-events to xenophobic attitudes via experienced daily satisfaction. Second, other criterion variables would be analyzed, for instance, physical or mental health. Concerning this, Junça-Silva & Silva, 2022a, 2022) demonstrated that neuroticism intensified the negative effects of uncertain environments on mental health. Thus, analyzing mental health within the AET framework would be relevant to deepen the understating of the interaction between neuroticism and situational characteristics (Junça-Silva, 2022; Junça-Silva et al., 2023). Third, there is evidence regarding the interaction of mindfulness with neuroticism (Decuypere et al., 2018). It would be relevant to explore whether mindfulness (trait or state) buffers the conditional negative effects of neuroticism regarding xenophobia or other criterion variables.

### Practical Implications

This study demonstrates the importance of neuroticism for xenophobic attitudes. Reducing neuroticism may be the key to reducing negative attitudes towards the out-group, such as xenophobic ones. Therefore, from a practical point of view, we must consider two purposes. First, neuroticism may be attenuated through other characteristics (mindfulness) (Decuypere et al., 2018; Junça-Silva & Silva, 2022a, 2022). Thus, designing and implementing mindfulness interventions may be a key factor to balance the negative effects of neuroticism.

Second, neuroticism may also be attenuated through training practices, for instance, training to reduce anxiety or to ameliorate cognitive appraisals of events (positive reappraisal approach) (e.g., Mizuno et al., 2022). These training practices could buffer the detrimental effects of neuroticism on xenophobic attitudes and acts (Hou et al., 2022).

## Conclusions

We can conclude that satisfaction is a consequence of daily micro-events, and at the same time is an antecedent of COVID-19 xenophobia. That is, a positive ratio of daily micro-events increases the levels of satisfaction, and in turn, COVID-19 xenophobic attitudes tend to decrease. However, this relationship is conditional upon the levels of individuals’ levels of neuroticism, as this moderates these positive effects. In other words, even if the day was good and satisfactory, neuroticism overshadows those effects, and hence, neurotic individuals tend to have more COVID-19 xenophobic attitudes. Thus, neuroticism might be understood as *the black unicorn effect*, once it enhances the blackness of the pandemic environment in which we are living. We may say that neuroticism threatens and blur the uplifting and satisfying nature of positive events, buffering its beneficial effect, and increasing the likelihood of these (neurotic) individuals engaging in xenophobic attitudes.
